# Supplementary material for: Leukaemia Inhibitory Factor (LIF) Inhibits Cancer Stem Cells Tumorigenic Properties through Hippo Kinases Activation in Gastric Cancer
Source: Cancers (Basel). 2020 Jul 22;12(8):2011. doi: 10.3390/cancers12082011 (PMC7464447; doi:10.3390/cancers12082011)
Supplement: Supplementary file 1 [file cancers-12-02011-s001.pdf]

Supplementary Materials: Leukaemia Inhibitory Factor (LIF) inhibits Cancer Stem Cells tumorigenic properties through Hippo kinases activation in Gastric Cancer

Lornella Seeneevassen, Julie Giraud, Silvia Molina-Castro, Elodie Sifré, Camille Tiffon, Clémentine Beauvoit, Cathy Staedel, Francis Mégraud, Philippe Lehours, Océane Martin, Hélène Boeuf, Pierre Dubus and Christine Varon

1. Proliferation Assay

AGS and MKN45 cells were plated (50,000 cells) in 24-well plates and treated or not, the day-after,with 50 ng/mL LIF for 48 h. Cells were then trypsinized and viable cells were counted using 0.2 mm depth Malassez counting chambers (PRECISS) in the presence of Trypan blue stain 0,4% (Gibco, ThermoFisher Scientific, Villebon sur Yvette, France).

2. Uptiblue Cell Viability Assay

Non-adherent 96-well cultured AGS tumourspheres were processed for cell viability analysis at day 10 after seeding. UptiBlue™ viable cell counting reagent (Interchim, Montluçon, France) (10 µL) was added to each well 2 h before analysis at λ *excl/em* : 570 / 590 nm which was carried out using a CLARIOstar® Plus fluorimeter (BMG Labtech, Ortenberg).

Table S1. List of primers used for RT-qPCR analysis.

Gene	Sequence Frame (5′-3′)	Sequence Reverse (5′-3′)
ALDH1A1	TCCTGGTTATGGGCCTACAG	CAACAGCATTGTCCAAGT
AREG	CGAACCACAAATACCTGGCTA	TCCATTTTTGCCTCCCTTTT
AXL	CGTAACCTCCACCTGGTCTC	TCCCATCGTCTGACAGCA
CD166	GCCTGGTTTCTGGGAGAACAC	GATGTTCATTTATCTTCCCTTTAAAGTTG
CD24	TTCTCCAAGCACCCAGCA	TGGAATAAATCTGCGTGGGTA
CD44	GGGCGTAACTCTGGAAGCA	TTAGCCCAGCTCACCTGAAAAT
c-FOS	CCGGGGATAGCCTCTCTTACT	CCAGGTCCGTGCAGAAGTC
CTGF	GCCACAAGCTGTCCAGTCTAATCG	TGCATTCTCCAGCCATCAAGAGAC
CYR61	ATGAATTGATTGCAGTTGGAAA	TAAAGGGTTGTATAGGATGCGA
EGR1	ACCTGACCGCAGAGTCTTTTC	GCCAGTATAGGTGATGGGGG
HPRT1	TGGTCAGGCAGTATAATCCA	GGTCCTTTTCACCAGCAAGCT
JUNB	TACCACGACGACTCATACACA	CGCTTTGAGACTCCGGTAGG
KLF5	CCTGGTCCAGACAAGATGTGA	GAACTGGTCTACGACTGAGGC
SOCS3	AGCTCGCATTCAGACTACCTA	AGTCGATCAGATGAACCACACT
TAZ	GTTTATGGGAGAGTCCGGGAG	AGT CTA AGG GCT TCG GCT CT
TBP	TGCACAGGAGCCAAGAGTGAA	CACATCACAGCTCCCCACCA

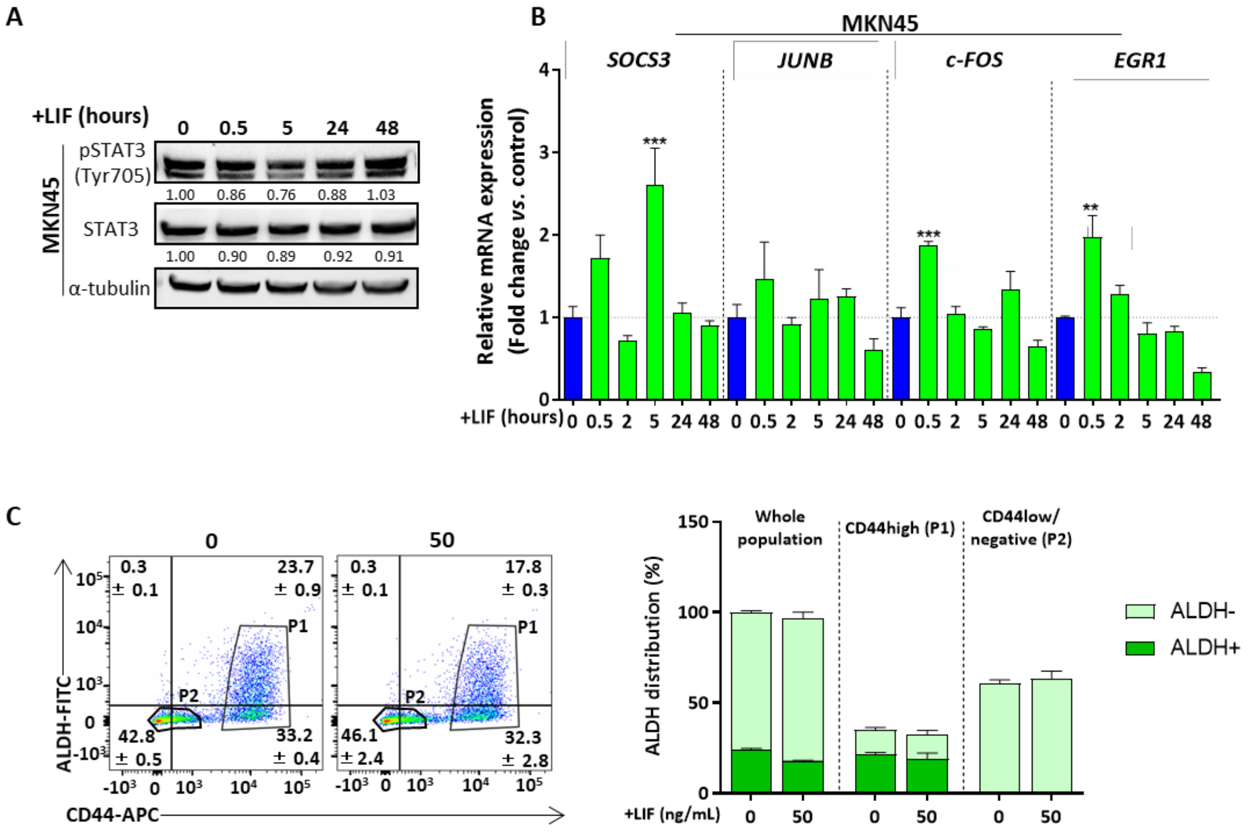

Figure S1. Effect of LIF treatment on JAKSTAT pathway and CSC markers expression in MKN45 cells. **(A)** p-STAT3^{Tyr705} and STAT3 protein levels in MKN45 cell line after treatment with 50 ng/mL LIF at different time intervals (0, 0.5, 5, 24, 48 h). Values under each band represent quantification of relative tubulin-normalised protein expression according to band density. **(B)** JAK/STAT targets relative mRNA expressions after treatment of MKN45 cells with (green) or without (blue) LIF at different tile intervals (0, 0.5, 2, 5, 24, 48 h). **(C)** Dot plot representation (left panel) and quantification (right panel) of flow cytometry analysis of gastric CSCs possessing cells distribution among CD44+/high (P1) and CD44-/low (P2) cells. Mean +/- SEM is represented in each quadrant of the dot plot graphs. $n = 3$, $^{*}p < 0.05$, $^{**}p < 0.005$, $^{***}p < 0.0005$ and $^{****}p < 0.0001$ *vs.* untreated controls with Mann-Whitney, Student t-tests and ANOVA statistical analyses.

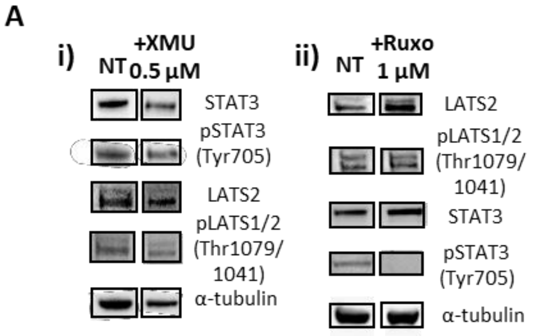

Figure S2. Effect of JAK1 inhibition and Hippo kinase inhibition on expression of members of the Hippo and JAKSTAT pathway. A) p-LATS1/2$^{Thr1079/1041}$, LATS2, p-STAT3^{Tyr705} and STAT3 protein levels in AGS cell line after 24 h treatment with 0.5 μM XMU-MP-1 (XMU) (i) or 1 μM Ruxolitinib (Ruxo) (ii).

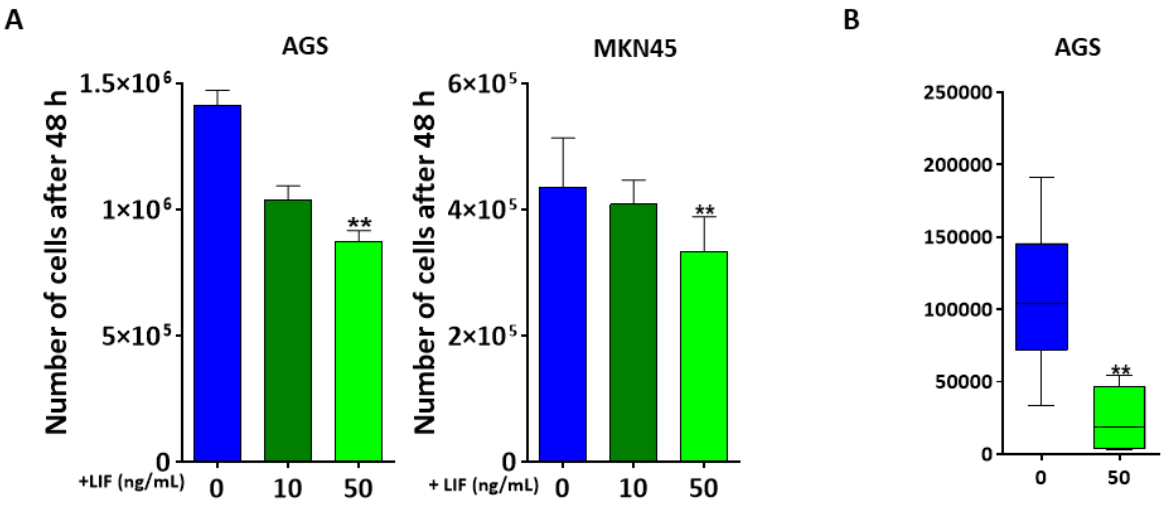

Figure S3. Effect of LIF treatment on proliferation and viability of gastric cancer cells. **(A)** Proliferation assay of AGS and MKN45 cells after 48 h treatment with different concentrations of LIF (0, 10 and 50 ng/mL). (**B)** Uptiblue-reagent based cell viability analysis of AGS tumourspheres treated (green), or not (blue) with 50 ng/mL LIF. For tumoursphere assays, LIF treatment was carried out every 48 h and sphere counting performed after 7 days. $n = 3$, *$p < 0.05$, **$p < 0.005$, ***$p < 0.0005$ and ****$p < 0.0001$ *vs.* untreated controls with Mann-Whitney and ANOVA statistical analyses.

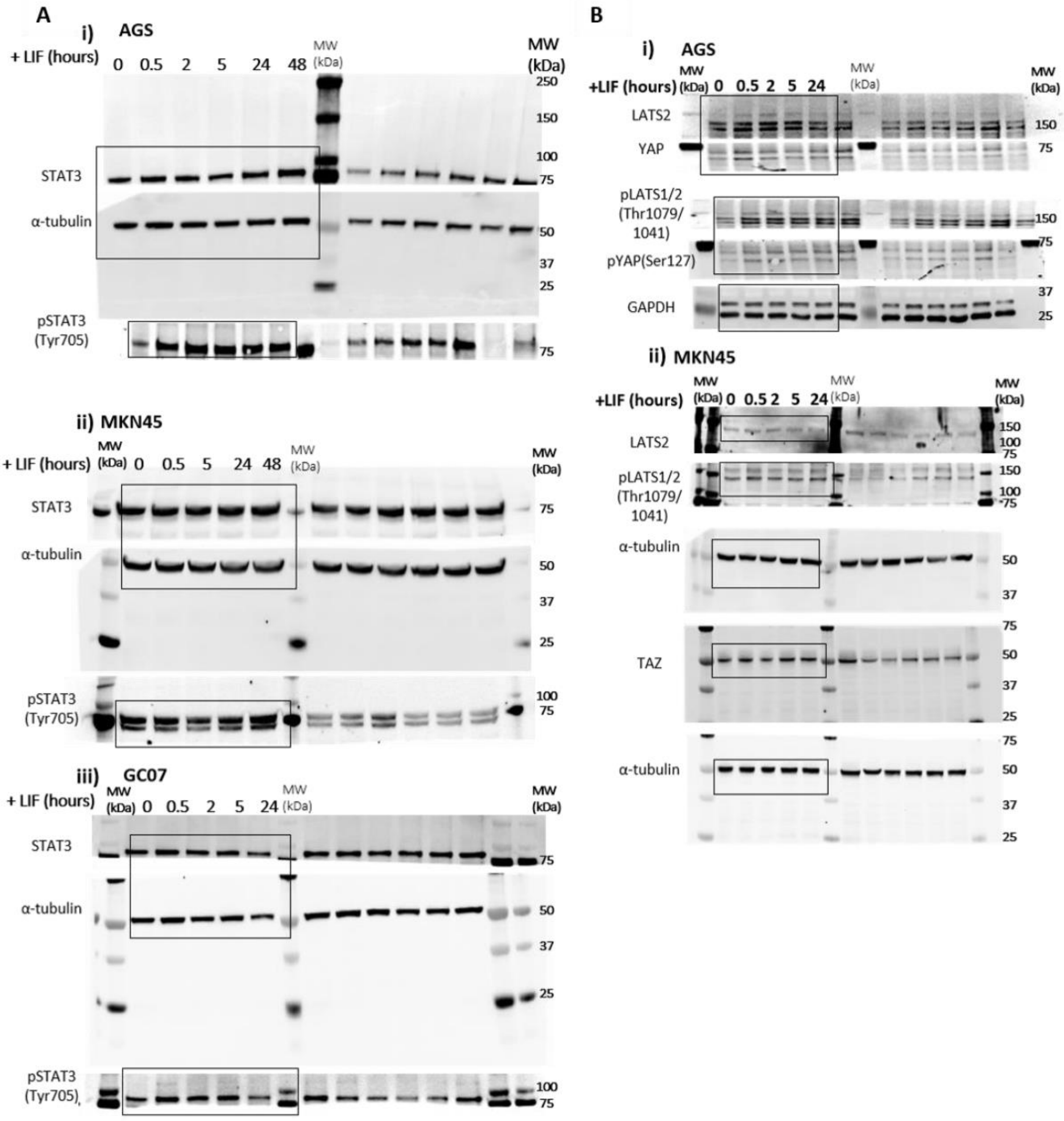

Figure S4: Raw Data of Figure 1A and 4A western blots: (**A**) Whole Figure 1A western 2 blots of i) AGS, ii) MKN45 and iii) GC07 cells. (**B**) Whole Figure 4A western blots of i) AGS and ii) 3 MKN45 cells. All blots were cut during process to allow separate incubation with the different 4 primary antibodies used.

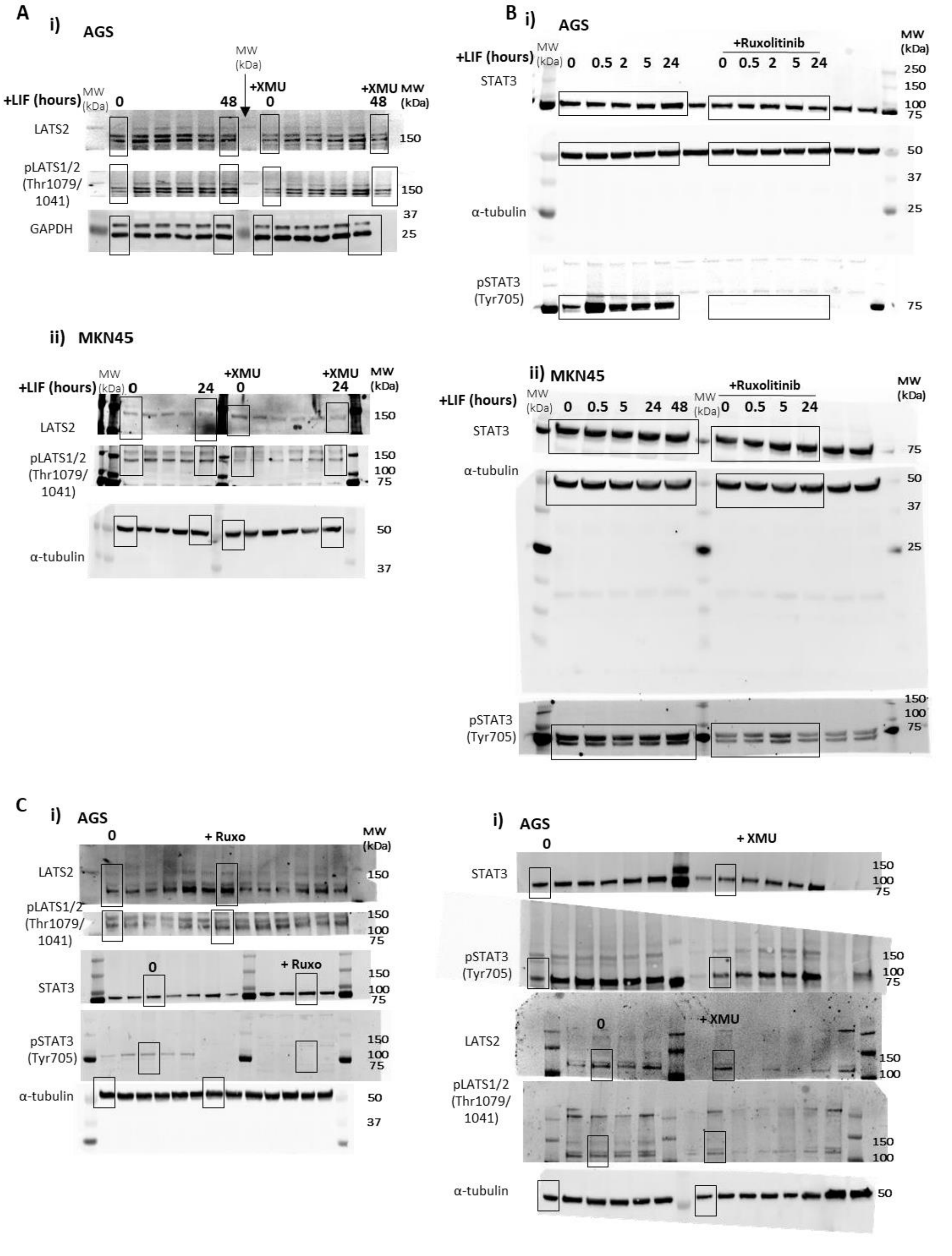

Figure S5. Raw Data of Figure 5 and Supplementary Figure S2 western blots: (**A**) Whole Figure 5A(i) western blots of i) AGS and ii) MKN45. (**B**) Whole Figure 5A(ii) western blots of i) AGS and ii) MKN45 cells. (**C**) Whole Figure S2 western blots of i) AGS with Ruxolitinib treatment (Ruxo) or ii) with XMU-MP-1 treatment (XMU). All blots were cut during process to allow separate incubation with the different primary antibodies used.

© 2020 by the authors. Licensee MDPI, Basel, Switzerland. This article is an open access article distributed under the terms and conditions of the Creative Commons Attribution (CC BY) license (http://creativecommons.org/licenses/by/4.0/).
